# P-1082. Antimicrobial Susceptibility of Enterobacterales Causing Infection in the Elderly: Focus on Aztreonam-avibactam and Recently Approved β-Lactamase Inhibitor Combinations

**DOI:** 10.1093/ofid/ofae631.1270

**Published:** 2025-01-29

**Authors:** Helio S Sader, Rodrigo E Mendes, Timothy Doyle, Marisa Winkler, Mariana Castanheira

**Affiliations:** JMI Laboratories, North Liberty, Iowa; JMI Laboratories, North Liberty, Iowa; Element Materials Technology/Jones Microbiology Institute, North Liberty, Iowa; Element Materials Technology/Jones Microbiology Institute, North Liberty, Iowa; JMI Laboratories, North Liberty, Iowa

## Abstract

**Background:**

Infections account for about 1/3 of all deaths in people 65 years and older and this population has a higher risk of exposure to antimicrobial-resistant bacteria. We evaluated the antimicrobial susceptibility of Enterobacterales (ENT) causing infection in elderly patients in United States (US) medical centers.

Activity of β-lactamase inhibitor combinations against Enterobacterales from elderly patients

**Figure:**
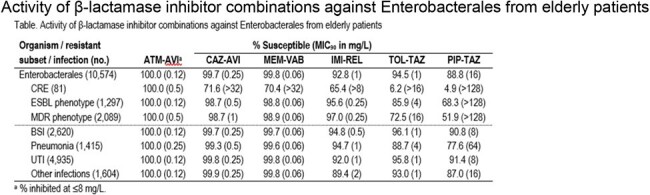

**Methods:**

Clinical isolates (1/patient) were consecutively collected from 72 US medical centers in 2021-2023 and susceptibility tested by broth microdilution. Results for 10,574 ENT isolates from elderly patients (≥65 years old) were analyzed and compared to 9,793 isolates from adult patients (age 18-64). Results were stratified by infection type and resistant subsets. Cefiderocol was only tested against carbapenem-resistant ENT (CRE). ATM-AVI was tested with AVI at fixed 4 mg/L and a PK/PD susceptible (S) breakpoint of ≤8 mg/L was applied for comparison. CRE isolates were screened for carbapenemases (CBase) by whole genome sequencing. Comparator agents included ceftazidime-avibactam (CAZ-AVI), meropenem-vaborbactam (MEM-VAB), imipenem-relebactam (IMI-REL), and cefiderocol (CRE only), among others.

**Results:**

All elderly isolates were inhibited at ATM-AVI MIC of ≤8 mg/L. CAZ-AVI (99.7% S) and MEM-VAB (99.8% S) were very active against ENT overall but exhibited limited activity against CRE (71.6% and 70.4% S, respectively; Table). The most active agents against CRE were ATM-AVI (100.0% inhibited at ≤8 mg/L) and cefiderocol (96.3% S). Multidrug-resistant (MDR; non-S to ≥3 classes) and ESBL phenotypes were observed in 19.8% and 12.3% of isolates, respectively, and ceftolozane-tazobactam (TOL-TAZ) and piperacillin-tazobactam (PIP-TAZ) showed limited activity against these organisms. Susceptibility to TOL-TAZ, PIP-TAZ, and ceftriaxone were lower among elderly isolates from pneumonia than BSI or UTI. The most common CBase were KPC (56.8% of CRE) and NDM (22.2%). A metallo-β-lactamase was identified in 24.7% of isolates. In general, susceptibility rates of isolates from the elderly were comparable (+/- < 2%) to those from the adult population.

**Conclusion:**

ATM-AVI demonstrated complete activity (100.0% S) against ENT causing infection in the elderly, including CRE and isolates with ESBL or MDR phenotypes.

**Disclosures:**

**Rodrigo E. Mendes, PhD**, GSK: Grant/Research Support **Marisa Winkler, MD, PhD**, Element Iowa City (JMI Laboratories) was contracted to perform services in 2023 for > 30 biotech and pharmaceutical companies: Grant/Research Support

